# Efficient and reproducible generation of human induced pluripotent stem cell-derived expandable liver organoids for disease modeling

**DOI:** 10.1038/s41598-023-50250-w

**Published:** 2023-12-22

**Authors:** Seon Ju Mun, Yeon-Hwa Hong, Yongbo Shin, Jaeseo Lee, Hyun-Soo Cho, Dae-Soo Kim, Kyung-Sook Chung, Myung Jin Son

**Affiliations:** 1 Stem Cell Convergence Research Center, Korea Research Institute of Bioscience and Biotechnology (KRIBB), 125 Gwahak-Ro, Yuseong-Gu, Daejeon, 34141 Republic of Korea; 2 Department of Functional Genomics, Korea University of Science & Technology (UST), 217 Gajungro, Yuseong-Gu, Daejeon, 34113 Republic of Korea; 3 Department of Bioinformatics, UST, 217 Gajungro, Yuseong-Gu, Daejeon, 34113 Republic of Korea; 4 Department of Digital Biotech Innovation Center, KRIBB, 125 Gwahak-Ro, Yuseong-Gu, Daejeon, 34141 Republic of Korea; 5 Biomedical Translational Research Center, KRIBB, 125 Gwahak-Ro, Yuseong-Gu, Daejeon, 34141 Republic of Korea

**Keywords:** Stem cells, Diseases

## Abstract

Genetic liver disease modeling is difficult because it is challenging to access patient tissue samples and to develop practical and relevant model systems. Previously, we developed novel proliferative and functional liver organoids from pluripotent stem cells; however, the protocol requires improvement for standardization and reproducible mass production. Here, we improved the method such that it is suitable for scalable expansion and relatively homogenous production, resulting in an efficient and reproducible process. Moreover, three medium components critical for long-term expansion were defined. Detailed transcriptome analysis revealed that fibroblast growth factor signaling, the essential pathway for hepatocyte proliferation during liver regeneration, was mainly enriched in proliferative liver organoids. Short hairpin RNA-mediated knockdown of *FGFR4* impaired the generation and proliferation of organoids. Finally, glycogen storage disease type Ia (GSD1a) patient-specific liver organoids were efficiently and reproducibly generated using the new protocol. They well maintained disease-specific phenotypes such as higher lipid and glycogen accumulation in the liver organoids and lactate secretion into the medium consistent with the main pathologic characteristics of patients with GSD1a. Therefore, our newly established liver organoid platform can provide scalable and practical personalized disease models and help to find new therapies for incurable liver diseases including genetic liver diseases.

## Introduction

It is difficult to study human liver development and liver disease progression, specifically congenital diseases, owing to a lack of relevant model systems. GSD1a, a hereditary rare disease, is caused by genetic mutations of an important enzyme in blood glucose homeostasis, glucose-6-phosphatase-α (G6Pase-α or G6PC1)^1^. Impaired hydrolysis of glucose-6-phosphate to glucose and phosphate due to deficiency of G6Pase enzyme activity results in severe hypoglycemia, and thus patients with GSD1a die in childhood without dietary therapies^2^. The inactivating mutation primarily targets gluconeogenic organs such as the liver, leading to hyperlipidemia and hepatomegaly followed by excessive hepatic glycogen accumulation. Long-term metabolic defects due to reduced gluconeogenesis and increased glycolysis and fatty acid synthesis lead to hepatocellular adenoma and, in severe cases, hepatocellular carcinoma^3^. However, no specific therapeutic options are available except liver transplantation.

As a fundamental approach, gene therapy is ongoing, but technical development is still needed to achieve efficiency with stable and long-term transgene expression^4^. However, relevant experimental model systems are limited due to low accessibility of liver tissue samples from patients who are children. Furthermore, primary human hepatocytes are challenging to maintain in long-term culture in the conventional 2D culture format^5^. As an alternative, stem cell-based organoid system has been developed to embody the structure and function of an actual organ^6^ and has been suggested to be the most advanced 3D liver model^7^. Liver organoids can be generated from both tissue-derived adult stem cells^8^ and pluripotent stem cells (PSCs)^9^. Given that GSD1a patients are infants and young children, PSC-derived organoids are beneficial because induced pluripotent stem cells (iPSCs) can provide unlimited patient-specific cell sources. Moreover, they can be a more appropriate system to model developmental biology and congenital disease progression because PSC-derived organoid generation protocols are based on the liver developmental process^10^.

Previously, we established a novel and practical liver organoid system that the PSC-derived liver organoids can be expanded beyond passage 90, are easily frozen and thawed, and are highly functional^11,12^. However, the protocol necessitated long-term differentiation and manual picking-up processes after the generation of the 3D cyst structure of the organoid. In addition, the medium components were undefined because a commercially available medium kit was used. Therefore, in this study, we developed an advanced method to generate liver organoids more efficiently and reproducibly and defined critical medium components for long-term expansion. Finally, we further demonstrated that GSD1a patient iPSC-derived liver organoids generated using this novel method maintain disease characteristics and provide a useful and personalized in vitro platform for modeling this liver disease.

## Results

### Improvement of the protocol for generating expandable human liver organoids from iPSCs

**Figure 1:**
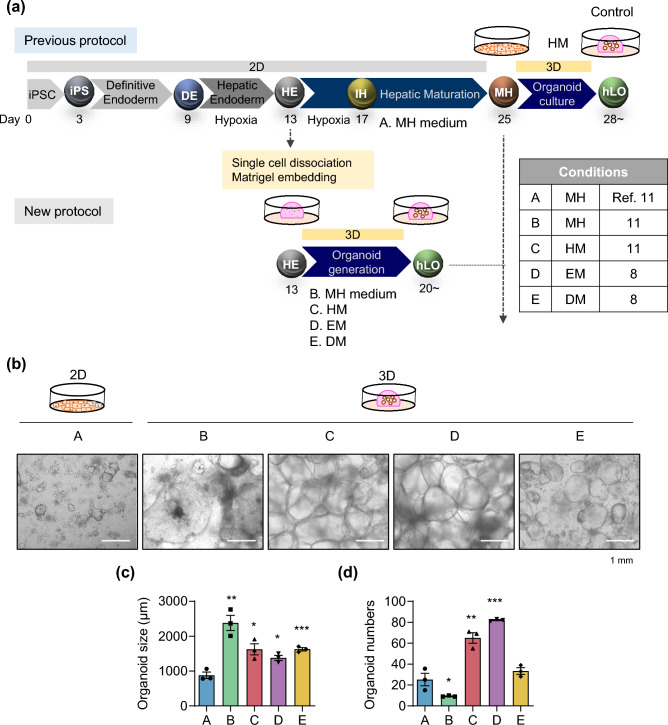
Efficient and reproducible generation of iPSC-derived liver organoids. **(a)** Schematic diagram of the previous (*upper*) and new (*lower*) protocols to generate iPSC-derived expandable liver organoids. A. MH (mature hepatocyte medium) (2D); B. MH (3D); C. HM (hepatic medium); D. EM (expansion medium); E. DM (differentiation medium). **(b)** Representative morphology of cells in each generation condition after 25 days of differentiation. **(c)** Organoid size and **(d)** organoid number in each condition after 25 days of differentiation. Data are the mean ± SEM (n = 3) and analyzed by the Student’s t-test. *p < 0.05, **p < 0.01, and ***p < 0.001.

Previously, we developed expandable liver organoids from PSCs following the stage-specific liver developmental process from PSCs to definitive endoderm (DE), hepatic endoderm (HE), immature hepatocytes (IH), and mature hepatocytes (MH)^11^ (Fig. 1a; Previous protocol). However, the protocol requires more than 3 weeks of differentiation in a 2D format, generation efficiencies are highly variable between batches, and a complicated manual picking up process is needed. Therefore, to advance and standardize the protocol, we moved the organoid generation step forward to the HE stage. Cells were dissociated into single cells after completing HE differentiation and then embedded into Matrigel as a 3D format (Fig. 1a; New protocol). From the HE stage, the new protocol takes only one week to generate organoids, whereas the previous protocol takes more than two weeks. At this stage, we compared the following four previously reported types of medium for liver organoid generation including those of Takebe and Taniguchi’s group^9^, Clevers’ group^8^, and ours^11^: MH (mature hepatocyte medium in ref. 11, modified by Takebe’s protocol^9^) (Fig. 1a; A and B), HM (hepatic medium in ref. 11) (C), EM (expansion medium in ref. 8) (D), and DM (differentiation medium in ref. 8) (E) (Supplementary Table S1). At 25 days after starting differentiation, cell morphologies were compared (Fig. 1b). At this time point, organoids were enlarged in all 3D groups than in the 2D control group (Fig. 1b,c). The number of organoids was increased by 2.6-fold in HM (C) and by 3.3-fold in EM (D) compared with the 2D control (A) (Fig. 1d).

### Characterization of organoids generated using the new protocol

**Figure 2:**
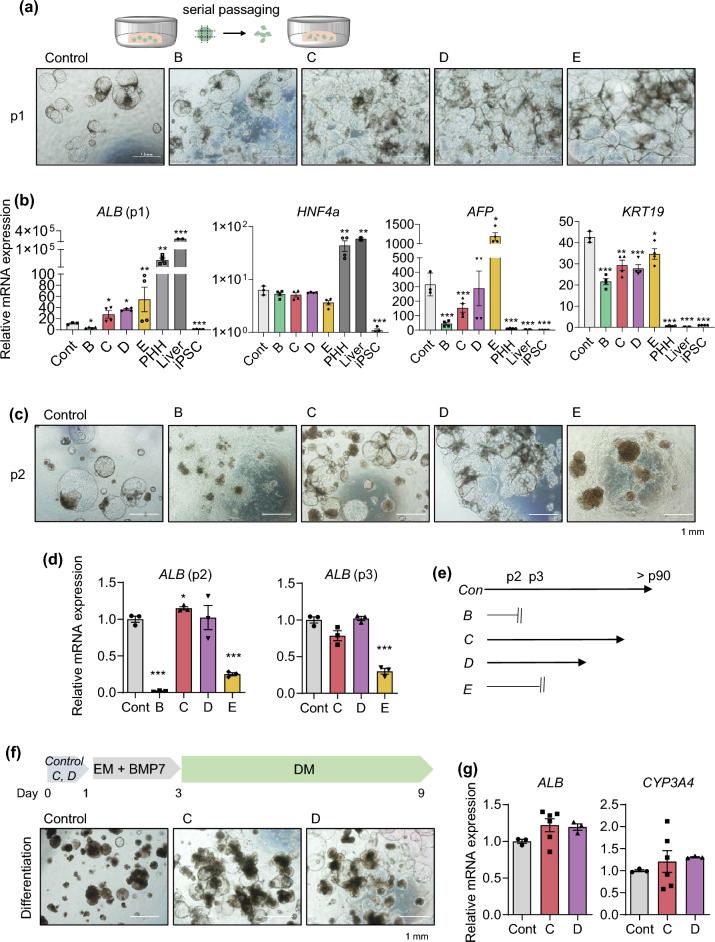
Characterization of organoids generated by the new protocol. **(a)** Representative morphology of organoids in each condition at passage 1 (p1). **(b)** mRNA expression levels of the indicated genes in each condition at p1. Primary human hepatocytes (PHH) and human adult liver tissue samples were used as positive controls and human iPSCs were used as a negative control. **(c)** Representative morphology of organoids in each condition at p2. **(d)** mRNA expression levels of *ALB* in each condition at p2 and p3. (**e)** Passages to which it was possible to expand in each condition. **(f)** Scheme of differentiation (*upper*) and representative morphology of the organoids after differentiation (*lower*). **(g)** mRNA expression levels of *ALB* and *CYP3A4* in each condition of the organoids after differentiation. Data are the mean ± SEM (n = 3) and analyzed by the Student’s t-test. *p < 0.05, **p < 0.01, and ***p < 0.001.

Next, to determine whether organoids generated under each condition could expand, we performed serial passaging by mechanically splitting the organoids using a blade and re-embedding them into fresh Matrigel (Fig. 2a). Albumin (*ALB)* gene expression was lowest in MH medium (B) and highest in DM medium (E) at passage 1 (Fig. 2b). However, *ALB* levels were significantly lower in both conditions (B and E) at passage 2 than in the control generated by the previous protocol (Fig. 2c,d). Importantly, further passaging was impossible beyond passage 2 in MH medium (B) and beyond passage 3 in DM medium (E) (Fig. 2c–e). Thus, further experiments could not be performed in either condition. On the other hand, organoids generated in HM (C) and EM (D) could be continuously passaged (Fig. 2c–e). Expression of hepatocyte markers such as *ALB* and hepatocyte nuclear factor 4 alpha (*HNF4A*) in both conditions was comparable with that in the control generated by the previous protocol (Fig. 2b,d). Their gene expression was substantially higher in the HM (C) and EM (D) conditions than in iPSCs, although it did not reach the levels observed in primary human hepatocytes (PHH) or human liver tissue (Fig. 2b). Expression of fetal liver/progenitor markers such as alpha-fetoprotein (*AFP*) and keratin 19 (*KRT19*) was rather lower in HM (C) and EM (D) than in the control generated by the previous protocol (Fig. 2b), indicating that earlier interaction with the 3D extracellular matrix can contribute to decrease the immaturity of iPSC-derived liver organoids. Moreover, expression of *AFP* was notably decreased under the HM (C) condition across passages and expression of the *KRT19* gradually decreased until passage 4 and remained consistent thereafter (Supplementary Fig. S1). Conversely, expression of the mature hepatocyte marker cytochrome P450 3A4 (*CYP3A4*) and the epithelial cell marker *EpCAM* significantly increased as the passages numbers increased (Supplementary Fig. S1). Furthermore, when organoids generated using HM (C) and EM (D) were further differentiated under the sequential EM and DM condition^8^ (Fig. 2f), mRNA expression of *ALB* and *CYP3A4* was compatible to that in the control (Fig. 2g). Like this, our previously defined novel medium, HM (C),^11^ is prominent in the potential of proliferation and differentiation without requiring expensive components such as R-spondin present in EM (D)^8^. Additionally, among 200 differentially expressed genes (DEGs, fold-change > 2, *p *value < 0.05) identified by RNA sequencing (RNA-seq) analysis comparing HM (C) and EM (D), 130 genes were up-regulated, and 70 genes were down-regulated (Supplementary Fig. S2a and Supplementary Table S2). Notably, genes associated with liver regeneration and proliferation, such as insulin like growth factor 2 (*IGF2),* insulin receptor substrate 2 (*IRS2)*, insulin receptor (*INSR),* CCAAT enhancer binding protein delta (*CEBPD)*^13^*,* and intercellular adhesion molecule 1 *(ICAM1)*^14,15^, were up-regulated. Furthermore, gene sets related to cholesterol metabolism, the androgen response, and glycolysis were notably enriched in the HM (C) condition compared with the EM (D) condition, according to Gene Set Enrichment Analysis (GSEA) (Supplementary Fig. S2b). These pathways are predominantly enriched in liver tissue^16–20^, suggesting that the HM condition contributes to the maintenance of self-renewal and hepatic maturation potential in liver organoids. Thus, we next determined which components of HM are essential for long-term expansion of liver organoids.

### Characterization of the components of HM required for long-term culture

**Figure 3:**
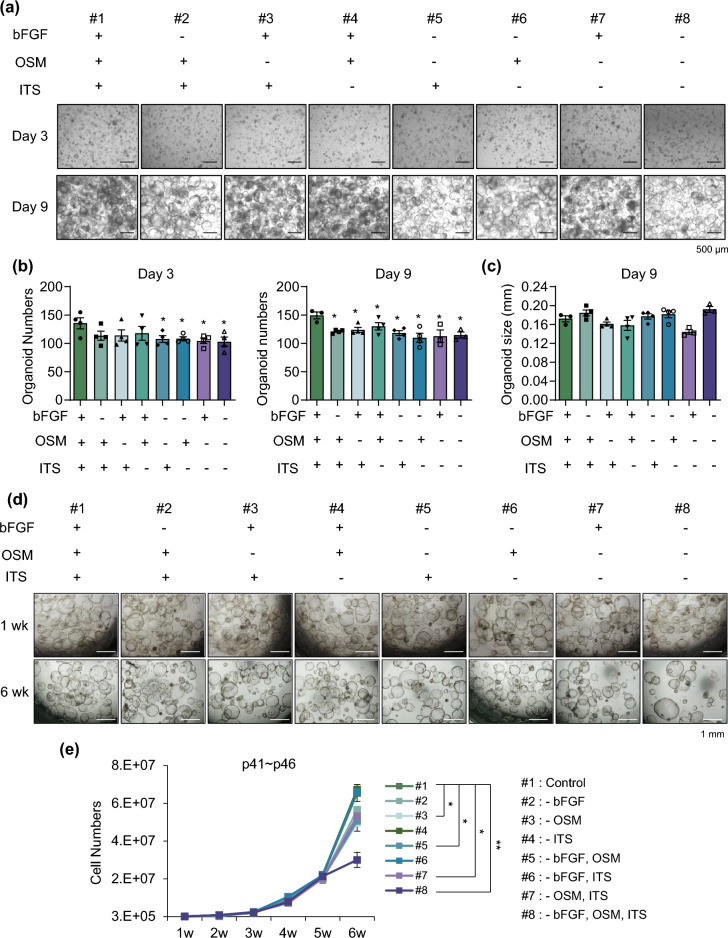
Essential factors in the medium for long-term culture. **(a)** Representative morphology of organoids in each condition on day 3 (*upper*) and day 9 (*lower*) after depletion. **(b)** Organoid number in each depleted condition on day 3 (*left*) and day 9 (*right*). **(c)** Organoid size in each depleted condition on day 9. **(d)** Representative morphology of late passage organoids in each condition at 1 week (*upper*) and 6 weeks (*lower*) after depletion. **(e)** Cell number of late passage organoids after serial passaging in each indicated depleted condition. Data are the mean ± SEM (n = 3) and analyzed by the Student’s t-test. *p < 0.05 and **p < 0.01.

As shown in Supplementary Table S1, basic fibroblast growth factor (bFGF), oncostatin M (OSM), and insulin-transferrin-selenium (ITS) are specific components of HM different from other medium. Therefore, we excluded each component individually and in combination from HM during organoid generation (Fig. 3a). The organoid number was slightly, but not significantly, decreased when only one factor was removed at 3 days after seeding (#2-#4), indicating that the other factors compensate during the early phase of organoid generation (Fig. 3b, *left*). However, when two (#5-#7) or all three (#8) factors were removed, the organoid number was decreased at this time point (Fig. 3b, *left*). At 9 days after seeding, the final organoid number was decreased in all groups (Fig. 3b, *right*). However, organoid size was not affected by elimination of bFGF, OSM, or ITS at 9 days after seeding, since after organoids were generated (Fig. 3c). Next, we determined the effect of removal of these factors on expansion of late passage organoids during long-term passaging (Fig. 3d). Organoids at passage 40 were expanded for 6 weeks without bFGF, OSM, or ITS. The cell number was significantly decreased in conditions #3 (-OSM), #5 (-bFGF, OSM), and #7 (-OSM, ITS) at 6 weeks of culture, and organoid proliferation was potent inhibited under condition #8, in which all three factors were removed (Fig. 3e). Interestingly, upon OSM removal, the population of smaller organoids significantly increased (Supplementary Fig. S3). Specifically, the percentage of small organoids (< 200 μm) was significantly increased in conditions #3, #5, #7, and #8. These data suggest that bFGF, OSM, and ITS in HM are essential for efficient generation and long-term expansion of organoids. Specifically, OSM plays a crucial role in the growth of organoids during late passages.

### Molecular characteristics of proliferative liver organoids

To define the molecular characteristics of proliferative liver organoids in HM, we compared their transcriptome profiles with those of non-proliferative cells in MH condition by RNA-seq (Supplementary Fig. S4). Top hallmark enriched pathways in HM compared with MH were mainly involved in two groups of gene sets: functional maturation (oxidative phosphorylation, fatty acid metabolism, and bile acid metabolism) and proliferation (E2F targets, MYC targets, and G2M check point) of organoids (Supplementary Fig. S4a). Representative top-ranked subsets corresponding to the two groups in GSEA are shown in Supplementary Fig, S4b. Next, DEGs whose expression was changed more than twice as much in HM compared with that in MH were selected (Supplementary Fig. S4c). The top ten ranked biological processes determined by gene ontology (GO) analysis included metabolic processes of lipids, organic acids, and monocarboxylic acids; homeostatic process; secretion; ion transport; and cell proliferation (Supplementary Fig. S4d). mRNA expression of representative genes corresponding to functional maturation was confirmed by real-time PCR (Supplementary Fig. S4e). Expression of genes related to lipid and bile acid metabolism was significantly higher in HM condition compared to MH (Supplementary Fig. S4e).

**Figure 4:**
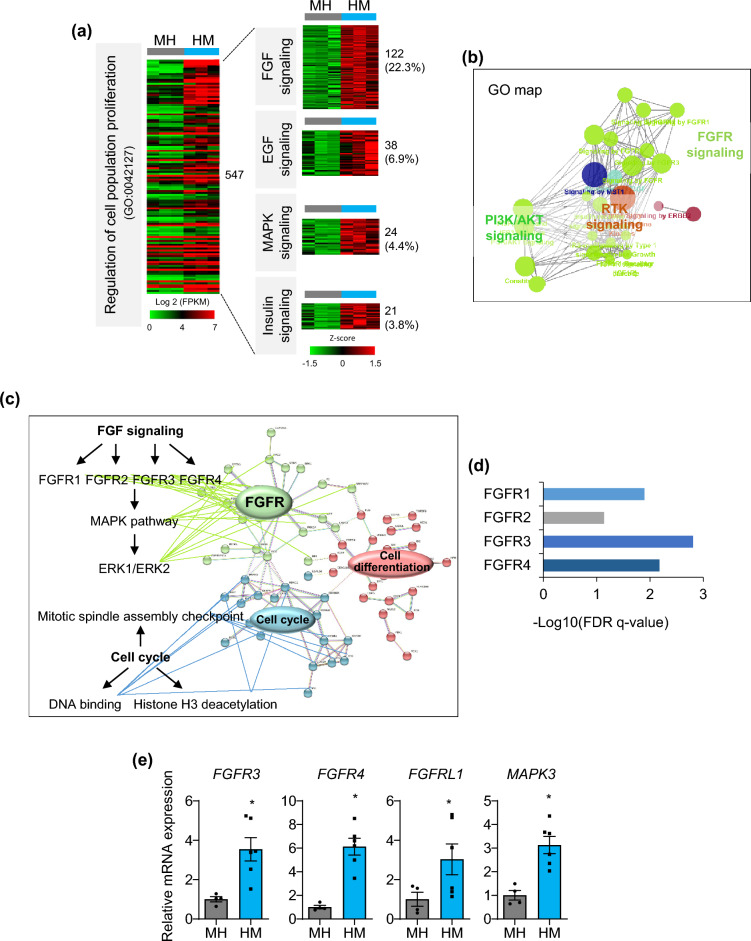
Top enriched pathways of proliferative liver organoids. **(a)** Heat map of the ‘regulation of cell population proliferation’ GO term (*left*) in DEGs between MH and HM and growth-related signaling within them (*right*). **(b)** GO map of ‘regulation of cell population proliferation’ genes enriched in HM. **(c)** PPI network analysis of 122 genes involved in FGF signaling. **(d)** Top enriched pathways in FGF signaling by Reactome analysis. **(e)** mRNA expression levels of genes related to major growth signaling in MH and HM condition. Data are the mean ± SEM (n = 3) and analyzed by the Student’s t-test. *p < 0.05.

Notably, proliferation-related genes (GO:0,042,127) accounted for approximately one-quarter of DEGs (547 of 2,142 DEGs (Supplementary Fig. S4c)) between MH and HM (Fig. 4a). Around 37% of them were associated with growth signaling such as FGF (22.3%), epidermal growth factor (EGF) (6.9%), mitogen-activated protein kinase (MAPK) (4.4%), and insulin (3.8%) signaling (Fig. 4a. Consistently, gene sets related to FGFR, Phosphoinositide 3-kinases (PI3K)/AKT, and receptor tyrosine kinases (RTK) signaling were enriched in the GO map (Fig. 4b). FGF signaling is the main pathway for hepatocyte proliferation in liver development and regeneration^21^, therefore, we analyzed the protein–protein interaction (PPI) network construction (Fig. 4c) and performed Reactome pathway analysis (Fig. 4d) focusing on FGF signaling (122 genes). High confidence interacting partners were FGFR signaling (FGFR, MAPK, and ERK pathways), regulation of the cell cycle (mitotic spindle assembly checkpoint, DNA binding, and histone H3 deacetylation), and cell differentiation (epithelial cell differentiation, bile duct development, and tube development) (Fig. 4c). In particular, FGFR3 and FGFR4 were highly enriched in HM condition (Fig. 4d). Real-time PCR confirmed that mRNA expression of *FGFR3*, *FGFR4*, *FGFRL1*, and the FGF signaling down-stream gene, *MAPK3*, was significantly increased in HM (Fig. 4e), indicating that FGFR signaling may be the major pathway that maintains the proliferative capacity of liver organoids in HM condition.

### Impaired generation and proliferation of liver organoids upon knockdown of FGFR4

**Figure 5:**
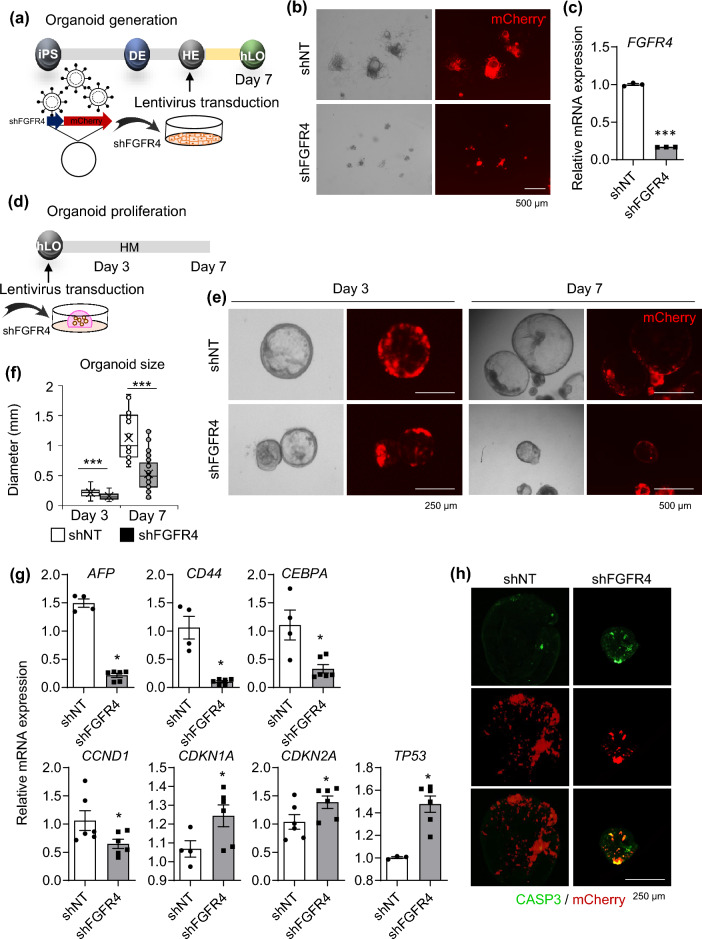
Effects of FGFR4 knockdown on generation and maintenance of organoids. **(a)** Schematic diagram of lentiviral transduction of shFGFR4 during organoid generation. **(b)** Representative morphology (*left*) and mCherry expression (*right*) of cells transduced with shNT (*upper*) and shFGFR4 (*lower*) at 7 days after transduction. **(c)** mRNA expression levels of *FGFR4* in shNT- and shFGFR4-transduced cells. **(d)** Schematic diagram of lentiviral transduction of shFGFR4 in HM-cultured proliferative organoids. **(e)** Representative morphology and mCherry expression of shNT- and shFGFR4-transduced organoids at 3 days (*left*) and 7 days (*right*) after transduction. **(f)** Organoid size in each condition at 3 and 7 days after transduction. **(g)** mRNA expression levels of the indicated genes in shNT- and shFGFR4-transduced cells at 3 days after knockdown. **(h)** Immunostaining of CASP3 (green, *upper*), mCherry signals (red, *middle*), and merged images (*bottom*) in each condition at 3 days after knockdown. Data are the mean ± SEM (n = 3) and analyzed by the Student’s t-test. *p < 0.05 and ***p < 0.001.

Next, we confirmed the effect of *FGFR4* knockdown by lentiviral short hairpin RNA (shRNA) transduction on the generation and proliferation of liver organoids (Fig. 5), because siRNA-mediated *Fgfr4* knockdown impairs hepatocyte proliferation in mice^21^. First, cells were dissociated into single cells at the HE stages during organoid generation and then transduced with shRNA targeting *FGFR4* (shFGFR4) or non-targeting control shRNA (shNT) (Fig. 5a). At 7 days after transduction, strong mCherry expression was detected in both groups, but shFGFR4-transduced cells failed to generate organoids (Fig. 5b and Supplementary Fig. S5a). *FGFR4* expression was downregulated in shFGFR4-transduced cells compared with shNT-transduced control (Fig. 5c). shFGFR4 transduction during organoid generation also significantly down-regulated mRNA expression of *FGFR4*-related genes such as *FGFR3*, *FGFRL1*, and *MAPK3* (Supplementary Fig. S5b); the cell cycle progression regulator, *CCND1*; and important factors for hepatic regeneration such as *AFP*, *LGR5*, *CD44*, *CEBPA*, and *PROM1* (Supplementary Fig. S5c). Second, HM-cultured proliferative organoids were transduced with shFGFR4 (Fig. 5d). Organoid growth was slow at 3 days after transduction and organoid size was prominently decreased at 7 days after transduction (Fig. 5e,f). shFGFR4 transduction potently downregulated mRNA expression of hepatic regeneration-related genes (*AFP*, *CD44*, and *CEBPA*) and *CCND1*, but upregulated expression of cell cycle inhibitor genes such as *CDKN1A*, *CDKN2A*, and *TP53* (Fig. 5g). Furthermore, caspase 3-positive apoptotic cells were prominently detected in organoids at 3 days after transduction of shFGFR4 (Fig. 5h). These results demonstrate that FGFR4 signaling is critical for the generation and maintenance of proliferative liver organoids.

Furthermore, we previously developed a Liver-specific Gene Expression Panel (LiGEP) that includes 93 genes expressed only in human adult liver tissue^22^ and used it as a validation platform to quantitatively evaluate the similarity between liver tissue and organoids generated from iPSCs^11^. Genes expressed in HM, but not in MH, were 20 from 93 genes (Supplementary Fig. S6a). These genes included those encoding the liver-specific glucose transporter, solute carrier family 2 member 2 (*SLC2A2*), drug-metabolizing enzymes (*CYPs* and *UGTs*), bile transport and synthesis enzymes (*SLCO1B1* and *BAAT*), and plasma proteins secreted by the liver (*SERPINA4*, *HABP2*, and *F12*). Importantly, genes encoding several regeneration-related factors such as *CYP8B1*, *SLC38A4*, *CXCL2*, and *TAT* were expressed specifically in HM. The mRNA expression levels of these enriched genes in HM were confirmed by real-time PCR (Supplementary Fig. S6b). Overall, these data demonstrate that liver organoids generated and cultured in HM condition may acquire the functional maturation capacity and regenerative and proliferative potential of the liver. Therefore, we next applied our novel organoid generation protocol to model the genetic liver disease GSD1a.

### Generation of liver organoids from GSD1a patient-derived iPSCs and their characterization

To acquire GSD1a patient-specific iPSCs, commercially available patient fibroblasts^23^ were reprogrammed using Sendai viruses (Supplementary Fig. S7a and b). Pluripotency of the established GSD1a patient-derived iPSCs was determined by alkaline phosphatase (AP) staining (Supplementary Fig. S7c) and by mRNA expression analysis (Supplementary Fig. S7d) and immunostaining (Supplementary Fig. S7e) of pluripotency markers. The differentiation potential was confirmed by immunostaining of markers of the three germ layers after in vitro differentiation through embryoid body (EB) formation (Supplementary Fig. S7f.). GSD1a patient-derived iPSCs maintained a normal karyotype after iPSC generation (Supplementary Fig. S7g).

**Figure 6:**
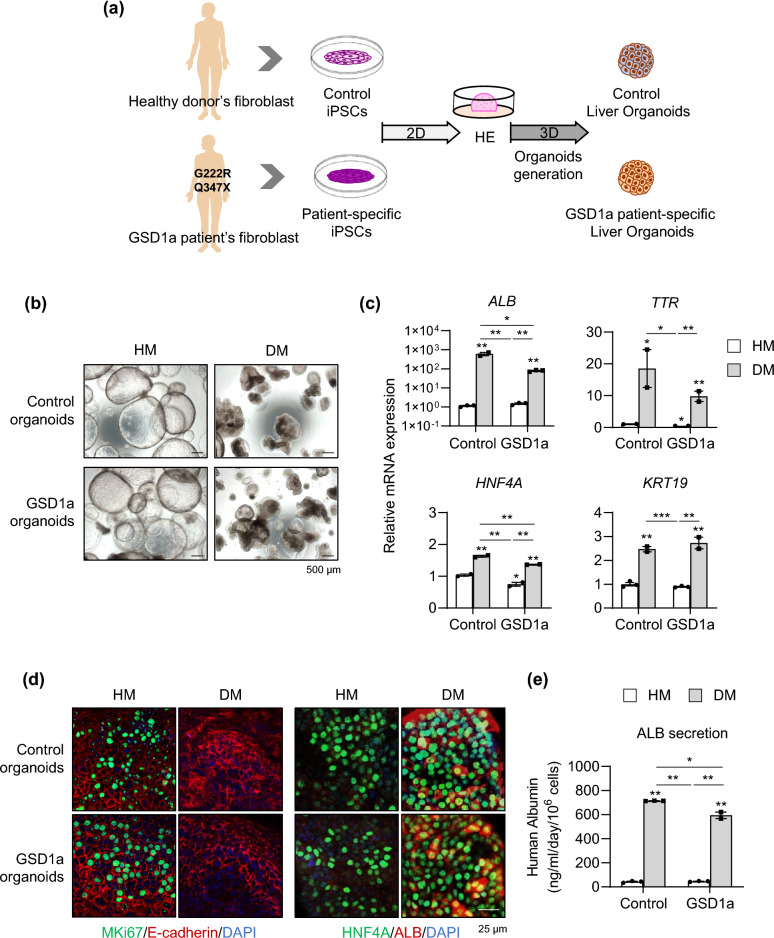
Generation of liver organoids from GSD1a patient-derived iPSCs by the new protocol and their characterization. **(a)** Schematic diagram of liver organoid generation from healthy donor control and GSD1a patient-derived iPSCs using the new protocol. **(b)** Representative morphology of control (*upper*) and GSD1a (*lower*) organoids in HM and DM condition. **(c)** mRNA expression levels of the indicated genes in control and GSD1a organoids in HM and DM conditions. **(d)** Immunostaining of control and GSD1a organoids in HM and DM condition with the indicated antibodies. **(e)** Quantification of ALB secretion by control and GSD1a organoids in HM and DM condition. Data are the mean ± SEM (n = 3) and analyzed by the Student’s t-test. *p < 0.05, **p < 0.01, and ***p < 0.001.

Next, liver organoids were generated from iPSCs of a healthy control and a GSD1a patient using the new protocol (Fig. 6a). GSD1a organoids were well generated and did not morphologically differ from control cells at each stage of differentiation during organoids generation (Supplementary Fig. S8a). Serial passaging was possible so far beyond passage 31 (Supplementary Fig. S8b), indicating the scalability and reproducibility of our new protocol. After 6 days of further differentiation in DM condition (Fig. 6b), GSD1a organoids had slightly lower levels of *ALB*, *TTR*, and *HNF4A* expression, but there were no remarkable differences compared with control organoids (Fig. 6c). MKI67-positive proliferating cells disappeared upon differentiation, and HNF4A and ALB protein expression (Fig. 6d) and ALB secretion (Fig. 6e) were prominently detected in both differentiated control and GSD1a organoids. These results demonstrate that liver organoids can be efficiently and reproducibly generated from GSD1a liver disease patient cells as well as healthy donor cells using the new protocol.

### Disease modeling of GSD1a using patient-specific liver organoids generated by the new protocol

**Figure 7:**
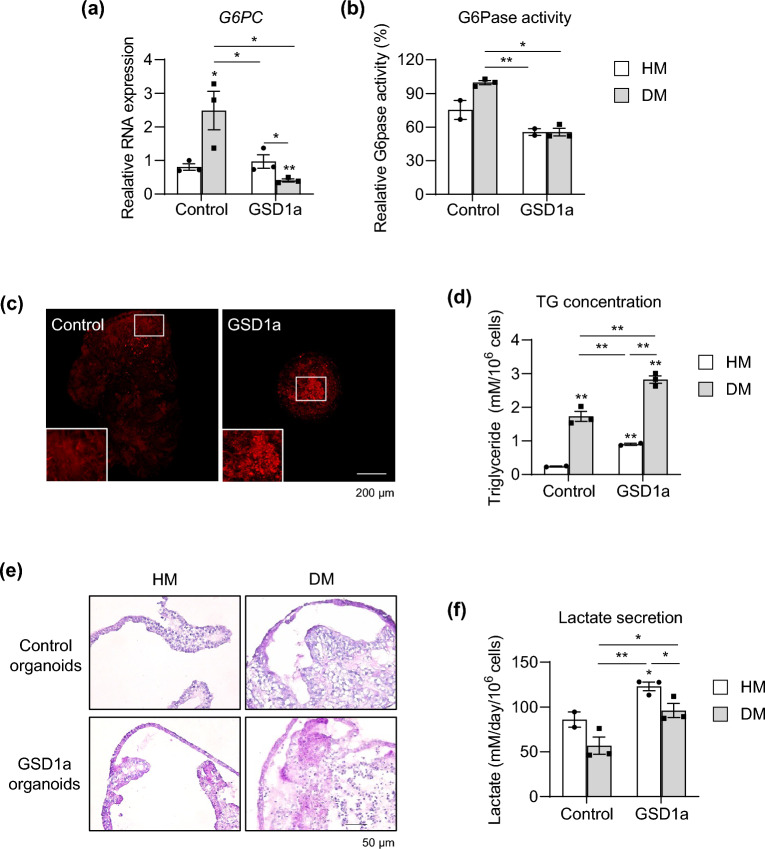
Disease modeling using GSD1a patient-specific liver organoids. **(a)** mRNA expression levels of *G6PC* in control and GSD1a organoids in HM and DM conditions. **(b)** G6Pase enzyme activity in control and GSD1a organoids in HM and DM condition. **(c)** Lipid droplets stained with Nile red in control and GSD1a organoids in DM condition. **(d)** Quantification of TG in control and GSD1a organoids in HM and DM condition. **(e)** Glycogen accumulation detected by PAS staining in control (*upper*) and GSD1a (*lower*) organoids in HM and DM conditions. **(f)** Quantification of lactate secretion by control and GSD1a organoids in HM and DM condition. Data are the mean ± SEM (n = 3) and analyzed by the Student’s t-test. *p < 0.05, **p < 0.01, and ***p < 0.001.

To characterize disease phenotypes of the generated patient iPSC-derived liver organoids, *G6PC* mRNA expression (Fig. 7a) and G6Pase enzyme activity (Fig. 7b) were determined. mRNA expression and enzyme activity were substantially decreased by 83.5% and 43%, respectively, in GSD1a organoids compared with control organoids in DM condition (Fig. 7a,b). Moreover, accumulated natural lipids detected by Nile red staining were distinctly observed in GSD1a organoids (Fig. 7c). Quantitatively, the triglyceride (TG) concentration was increased over 1.9-fold in GSD1a organoids compared with that in control organoids in DM condition (Fig. 7d). In addition, periodic acid-Schiff (PAS) staining revealed that glycogen strongly accumulated in GSD1a organoids (Fig. 7e). Moreover, increased lactate secretion of GSD1a organoids was easily detected even in medium both from HM- and DM-cultured organoids (Fig. 7f). Taken together, these results demonstrate that liver organoids generated by the new protocol well maintain patient-specific disease phenotypes, and their scalability and practicality may facilitate their application as an organoid platform for personalized disease modeling and drug screening for treatments of liver diseases.

## Discussion

Liver organoids are the most advanced 3D liver model to preserve the pathologic characteristics of patients with various liver diseases. However, the scalability and standardization of their generation protocol are major challenges in the organoid field^24^. In this study, we improved and simplified our previous protocol^11^ to make it reproducible and suitable for mass production. We also examined transcriptomic profiles in detail and showed that FGF/R signaling is a major pathway for expansion and long-term culture of liver organoids. Finally, expandable and functional liver organoids generated using the new protocol can sufficiently provide human liver models for studying inherited liver diseases and finding new therapies for these diseases.

Our new protocol reproducibly generated liver organoids regardless of the somatic cell origin of iPSCs e.g., cord blood, bone marrow, and urine (data not shown). In addition, liver organoid generation was not affected by reprogramming methods such as Sendai viruses and episomal vectors, and was easily performed using human embryonic stem cells as well as iPSCs (data not shown). The morphology of our HM-cultured organoids closely resembles that of liver organoids derived from iPSCs by Vallier's group^25^. However, the liver organoids generated by Vallier’s group predominantly consist of cholangiocytes, while our liver organoids have a diverse cellular composition, encompassing hepatocytes, cholangiocytes, and non-parenchymal cell populations, including stellate cells and immune cells^26^. Notably, our organoids include ALB-positive cells, which constituted 38.63% of cells in the HM condition^11^, while Vallier's group reported a percentage of 15.89%^25^, difference of more than two-fold. Furthermore, upon further differentiation of our organoids under the DM condition, the ALB-positive cell population significantly increased to 79.44%^11^, closely resembling the hepatocyte population found in the liver. Consequently, the organoids generated by Vallier's group may serve as a valuable cholangiocyte-oriented model, while our multicellular liver organoids offer versatility for various applications, such as studying disease progression mediated by immune cells or stellate cells.

Critical components for the long-term expansion of our liver organoids were identified as bFGF, OSM, and ITS. bFGF is one of the primary signals for foregut endoderm patterning derived from the cardiac mesoderm, which is essential for hepatic progenitor specification^27^. OSM belongs to the interleukin-6 (IL-6) family secreted by hematopoietic cells in fetal liver, boosting hepatocyte proliferation and maturation of fetal hepatocytes^28–31^. Upon removal of OSM, organoid proliferation substantially decreased (Fig. 3e) and the population of smaller organoids significantly increased (Supplementary Fig. S3), indicating that OSM is the strongest contributor to organoid expansion. Additionally, upregulated genes in the HM condition, such as *CEBPD* and *ICAM* (Supplementary Fig. S2a and Supplementary Table S2), stimulated by IL-6, have been reported to participate in early liver development and adult liver regeneration^14,15,32^. ITS is generally used as a chemically defined supplement to support survival of hepatocytes during hepatic differentiation^33^. Genes related to insulin signaling, such as *IGF2, IRS2*, and *INSR,* were also upregulated in the HM condition (Supplementary Fig. S2a and Supplementary Table S2). Overall, the coordinated functions of these factors may simultaneously promote functional maturation and proliferation of liver organoids.

FGF/FGFR signaling was the major pathway for regulation of cell proliferation enriched in organoids in HM (Fig. 4). FGFR3 signaling, which was highly increased in organoids in HM according to Reactome analysis (Fig. 4d), is important for hepatocyte survival. Loss of Fgfr3 increases tissue damage after liver injury, suggesting that Fgfr3 has a cytoprotective effect in hepatocytes^34^. Moreover, Fgfr4-deficient mice exhibit impaired hepatocyte proliferation following partial hepatectomy^21,35^. Therefore, augmented FGF/FGFR signaling in HM condition may be critical for long-term expansion of liver organoids. This was experimentally confirmed by shRNA-mediated knockdown of FGFR4 in our organoid system (Fig. 5 and Supplementary Fig. S5). Importantly, FGF can be a cost-effective substitute for the expensive Wnt agonist, R-spondin. In LiGEP analysis^22^, regeneration-related factors such as *CYP8B1*, *SLC38A4*, *CXCL2*, *TAT*, and *AFM* were encountered in HM compared with MH condition (Supplementary Fig. S6). Importantly, liver organoids generated from GSD1a patient-derived iPSCs by the new protocol exhibited disease-specific phenotypes, highlighting the potential of these organoids for congenital liver disease modeling and drug screening. The longstanding functional maintenance is advantageous for simulating long-term complications of disease progression^36^, which is challenging to achieve using conventional liver models.

## Methods

### Cells

Human foreskin fibroblasts (CRL-2097) were purchased from the American Type Culture Collection and used as a healthy donor control. Fibroblasts from a 25-year-old male patient with GSD1a were purchased from the Coriell Institute^23^.

### iPSC generation

The GSD1a patient’s fibroblasts were reprogrammed using a CytoTune®-iPS 2.0 Sendai Reprogramming Kit (Thermo Fisher) as previously described^11^. The iPSC colonies were picked and maintained on a γ-irradiated mouse embryonic fibroblast feeder layer in DMEM/F-12 medium (Thermo Fisher) containing 1% non-essential amino acids (Thermo Fisher), 1% GlutaMax I (Thermo Fisher), 0.1 mM β-mercaptoethanol (Thermo Fisher), 20% KnockOut™ Serum Replacement (SR, Thermo Fisher), 10 ng/ml bFGF, (PeproTech), and 1% penicillin–streptomycin (Thermo Fisher). iPSCs were passaged weekly with collagenase type IV (Thermo Fisher).

### Organoid generation

The previous organoid generation protocol was described in detail in our recent work^11^. Here, the protocol was modified such that cells were detached and dissociated into single cells using TrypLE™ Express Enzyme (Thermo Fisher) after HE induction at day 13 of differentiation. Dissociated single cells were embedded into Matrigel (Corning) with HM, and 10 μM Y-27632 (Tocris) was added for 3 days to improve viability. Three-dimensional liver organoids formed in 3–5 days.

### Organoid culture

The generated organoids were routinely divided at a ratio of 1:5–10 and re-embedded into fresh Matrigel to save space. After 1 week, fully enlarged organoids were mechanically split with surgical blade and then passaged weekly at a ratio of 1:3–1:5. Split organoids were also cryopreserved with mFreSR™ (Stem Cell Technology) for long-term storage. For further differentiation of organoids, HM-cultured organoids were incubated in EM supplemented with 25 ng/ml BMP7 (PeproTech) for 3 days and then in differentiation medium DM for an additional 6 days. The medium composition is described in detail in Supplementary Table S1.

### RNA extraction and real-time PCR

Total RNA was extracted using easy-BLUE™ reagent (iNtRON) according to the manufacturer’s instructions. Complementary DNA was synthesized using TOPscript™ RT DryMIX (Enzynomics) and quantitative PCR was performed using Fast SYBR® Green Master Mix (Applied Biosystems) with gene-specific primers (Supplementary Table S3). *ACTB* was used as an internal control.

### RNA-seq and transcriptome analysis

RNA-seq was performed with an Illumina HiSeq 2500 instrument and a quality check was completed using FastQC, Cutadapt (v1.13), and Sickle (v1.33). FPKM (fragments per kilobase of transcript per million mapped reads) values were used to quantify gene expression levels. Transcripts of 25,270 genes were included in GSEAs. A heat map was generated using MeV 4.9.0 software. DEGs whose expression levels were changed more than twice as much in HM compared with those in MH or EM and with a *p *value less than 0.05 were selected. FGF signaling genes were submitted to the Search Tool for the Retrieval of Interacting Gene/Proteins (STRING) database for PPI network construction. Reactome analysis was performed using the Reactome Pathway database (https://reactome.org), and results were visualized using Cytoscape software.

### Immunocytochemistry

Organoids were fixed with 4% paraformaldehyde (PFA, Biosesang) for 30 min at room temperature (RT), washed with phosphate-buffered saline (PBS, Welgene) and then incubated with specific primary antibodies at 4 ℃ overnight (Supplementary Table S4). Organoids were washed with PBS containing 0.05% Tween-20 (Sigma-Aldrich) and incubated with Alexa Fluor®-conjugated secondary antibodies for 50 min at RT in the dark. Nuclei were stained with 4′,6-diamidino-2-phenylindole (DAPI, Sigma-Aldrich) and mounting solution (Dako) was added. Images were obtained using an Olympus microscope.

### Transduction of lentiviruses

HE cells were dissociated into single cells or liver organoids were split into small pieces using a blade. Cells or split organoids were transduced with lentiviral particles (Vectorbuilder) at a multiplicity of infection of 5 and incubated overnight with 8 μg/ml polybrene (Vectorbuilder), 2% Matrigel, and 10 μM Y-27632 in ultra-low attachment plates (Corning). The next day, transduced cells or organoids were embedded in 30 μl of Matrigel.

### Quantification of ALB secretion, G6Pase activity, the TG concentration, and lactate secretion

To quantify ALB secretion, the culture supernatant was collected after 24 h of culture and assayed using a Human Albumin ELISA Kit (Bethyl Laboratories) following the manufacturer’s instructions. Absorbance was measured using a Spectra Max M3 microplate reader (Molecular Devices). To quantify G6Pase enzyme activity, organoids were lysed with ice-cold G6Pase buffer, and the supernatant obtained after centrifugation at 4 °C for 10 min was analyzed using a G6Pase enzyme activity assay kit (Biomedical Research Service Center). Absorbance at 660 nm was measured using a Spectra Max M3 microplate reader. To quantify TG concentration, organoids were sonicated with 5% NP-40 (Sigma-Aldrich) solution and boiled at 100 °C for 5 min. The supernatant obtained after centrifugation was diluted tenfold and analyzed using a TG assay kit (Abcam). Absorbance was measured using a microplate reader. To quantify lactate secretion, the culture supernatant was harvested after 24 h of organoid incubation and assayed using lactate colorimetric assay kit (Biovision) according to the manufacturer’s protocols. Absorbance at 450 nm was measured using a microplate reader.

### Nile red, PAS, and AP staining

For Nile red staining, organoids were washed with cold PBS and fixed with 4% PFA for 15 min at RT. The organoids were stained with 10 µg/ml Nile red solution (Thermo Fisher) for 5 min at RT in the dark. Images were obtained using a Zeiss confocal microscope. For PAS staining, PFA-fixed organoids were washed with PBS, and then incubated with 30% sucrose solution at 4 °C overnight. Dehydrated organoids were embedded in O.C.T. compound (Sakura Finetek) and snap frozen. Sections were obtained with a cryostat (LEICA) as an 8 μm thick and stained with Periodic acid solution for 5 min, Schiff reagent for 15 min, and Mayer’s hematoxylin solution for 30 s at RT using a PAS staining kit (IHC WORLD). Images were obtained using an Olympus microscope. For AP staining, iPSCs were fixed in citrate–acetone–formaldehyde solution and then stained with AP staining solution (Sigma-Aldrich). Cell images were captured using an Olympus microscope.

### In vitro differentiation assay

For EB formation, GSD1a patient-derived iPSCs were detached with collagenase type IV and Dispase (Thermo Fisher) for 10 min at 37 ℃. Dissociated iPSC colonies were washed with basal media and cultured with 10% SR in suspension for 4 days in a low-attachment 35-mm Petri dish (SPL Life science). Aggregated EBs were attached to a Matrigel-coated plate and then spontaneously differentiated in basal medium supplemented with 10% fetal bovine serum (FBS, Thermo Fisher) for 2 weeks.

### Karyotype analysis

iPSCs were cultured in a Matrigel-coated T25 flask using a mTeSR™1 Complete Kit (Stem Cell Technology) for 3–4 days. Chromosomal GTG banding karyotype analysis was performed at 550 resolution by GenDix, Inc.

### Statistical analysis

Data analysis was performed using the GraphPad Prism 8 software package (GraphPad Software, San Diego, CA, USA). All data are from more than three independent biological replicates. Graphs show the mean ± SEM of triplicate samples. The Student’s t test was used to perform the inter-group comparisons and p < 0.05 indicated statistical significance.

### Ethics approval and consent to participate

The study was approved by the Institutional Review Board (IRB) at the Public Institutional Bioethics Committee designated by the South Korea Ministry of Health and Welfare (Title: Study of generation of human induced pluripotent stem cells and their differentiation to target cells, IRB file no: P01-201609–31-002, date: September 2021).

### Supplementary Information

Supplementary Information.

## Data Availability

All data generated or analyzed during this study are included in this published article and its supplementary information files. The data that support the findings of this study are available from the corresponding author upon reasonable request. Gene expression data are available at SRA, accession number PRJNA528522 (https://www.ncbi.nlm.nih.gov/bioproject/PRJNA528 522/).
