# Supplementary material for: Efficient and reproducible generation of human induced pluripotent stem cell-derived expandable liver organoids for disease modeling
Source: Sci Rep. 2023 Dec 22;13:22935. doi: 10.1038/s41598-023-50250-w (PMC10739970; doi:10.1038/s41598-023-50250-w)
Supplement: Supplementary file 1 — Supplementary Information. [file 41598_2023_50250_MOESM1_ESM.pdf]

Supplemental Figure 1

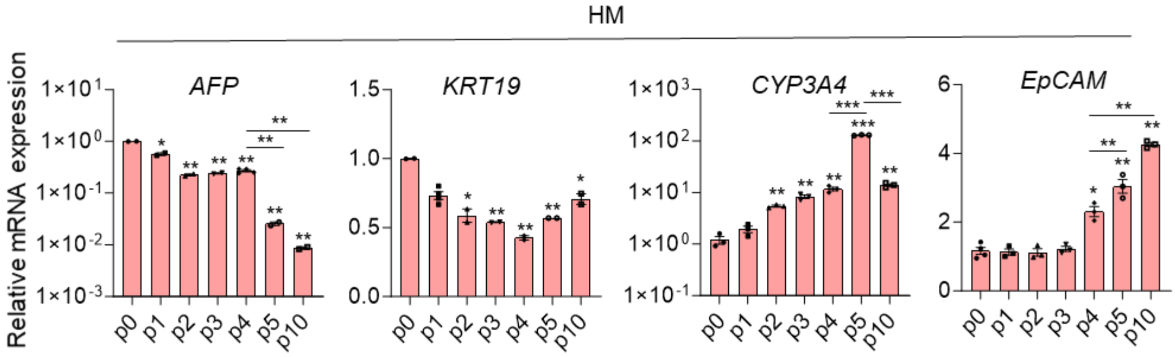

Supplemental Figure 2

(a)

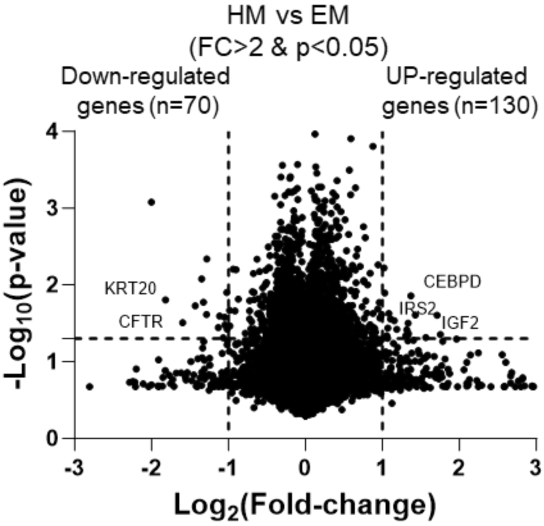

(b)

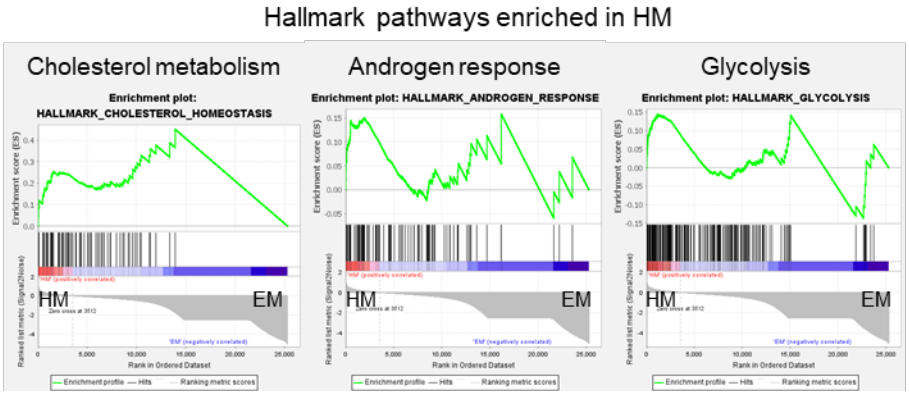

Supplemental Figure 3

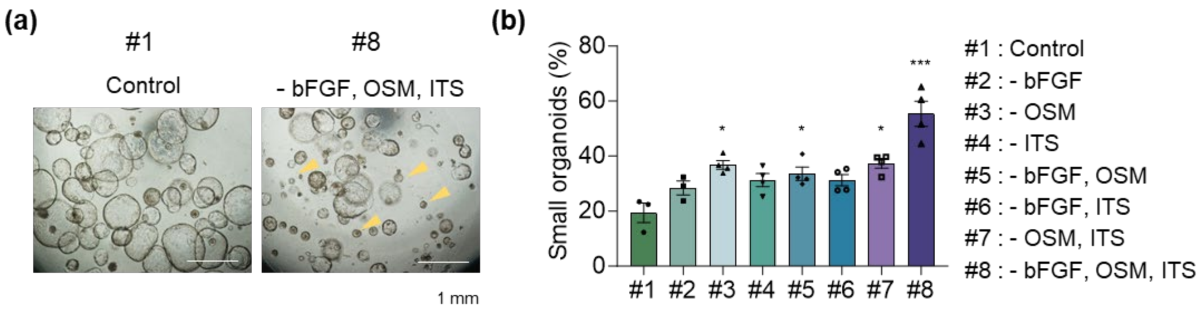

Supplemental Figure 4

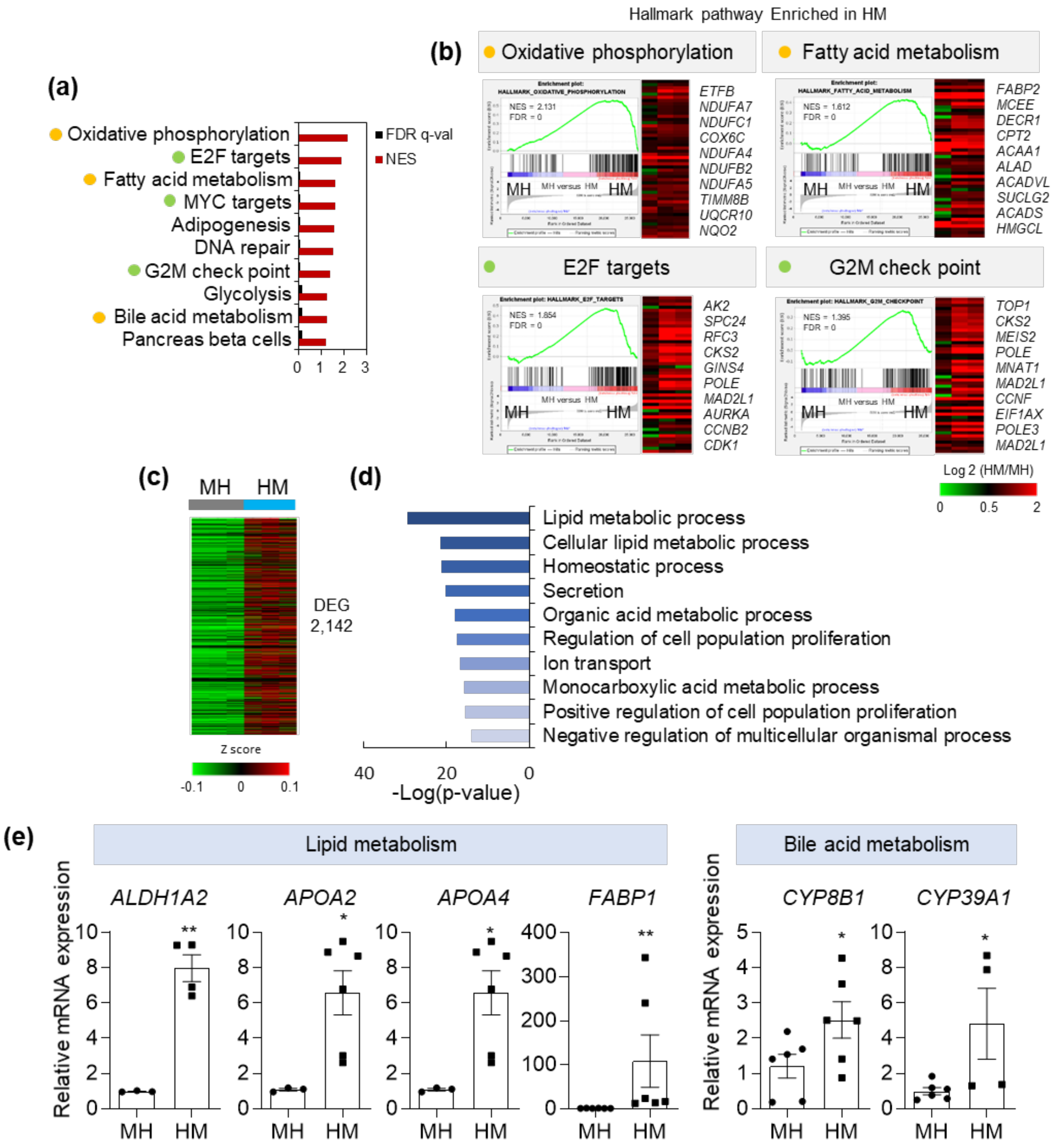

Supplemental Figure 5

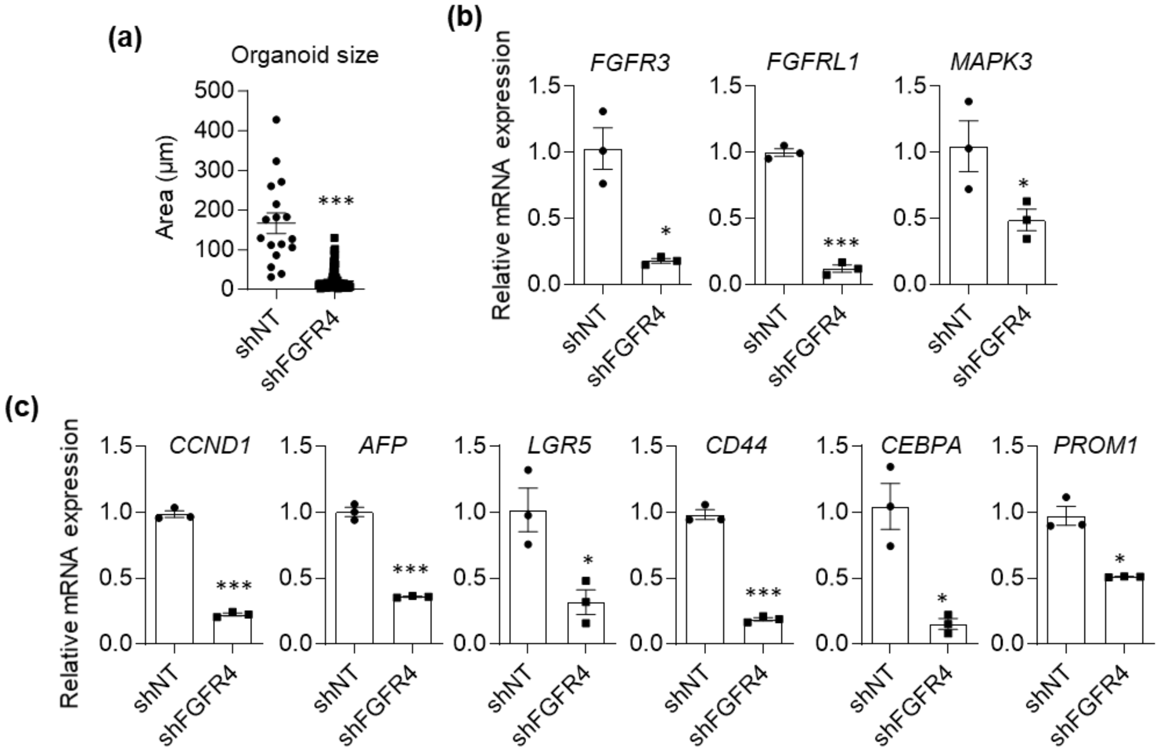

Supplemental Figure 6

(a)

Liver-specific Gene Expression Panel (LiGEP)

	Symbol	MH	HM	Liver
1	SLC2A2			
2	CYP2C9			
3	CYP2C8			
4	CYP8B1			
5	UGT2B15			
6	UGT2B10			
7	AKR1C4			
8	SLC38A4			
9	CXCL2			
10	TAT			
11	SLCO1B1			
12	BAAT			
13	SERPINA4			
14	HABP2			
15	F12			
16	CPB2			
17	SERPINA6			
18	GC			
19	CFHR3			
20	APCS			

(b)

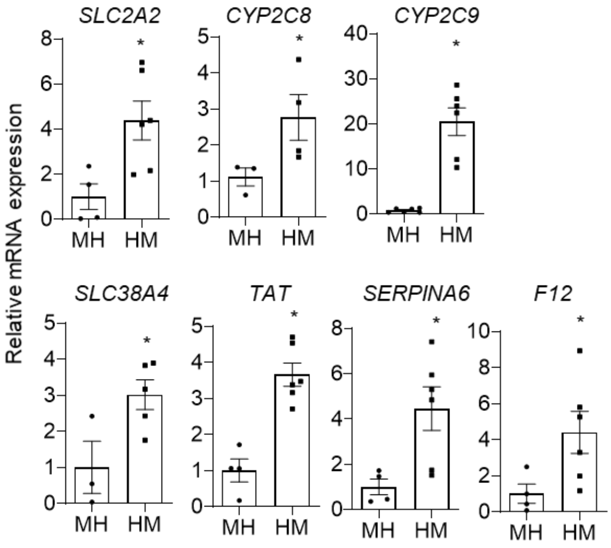

Supplemental Figure 7

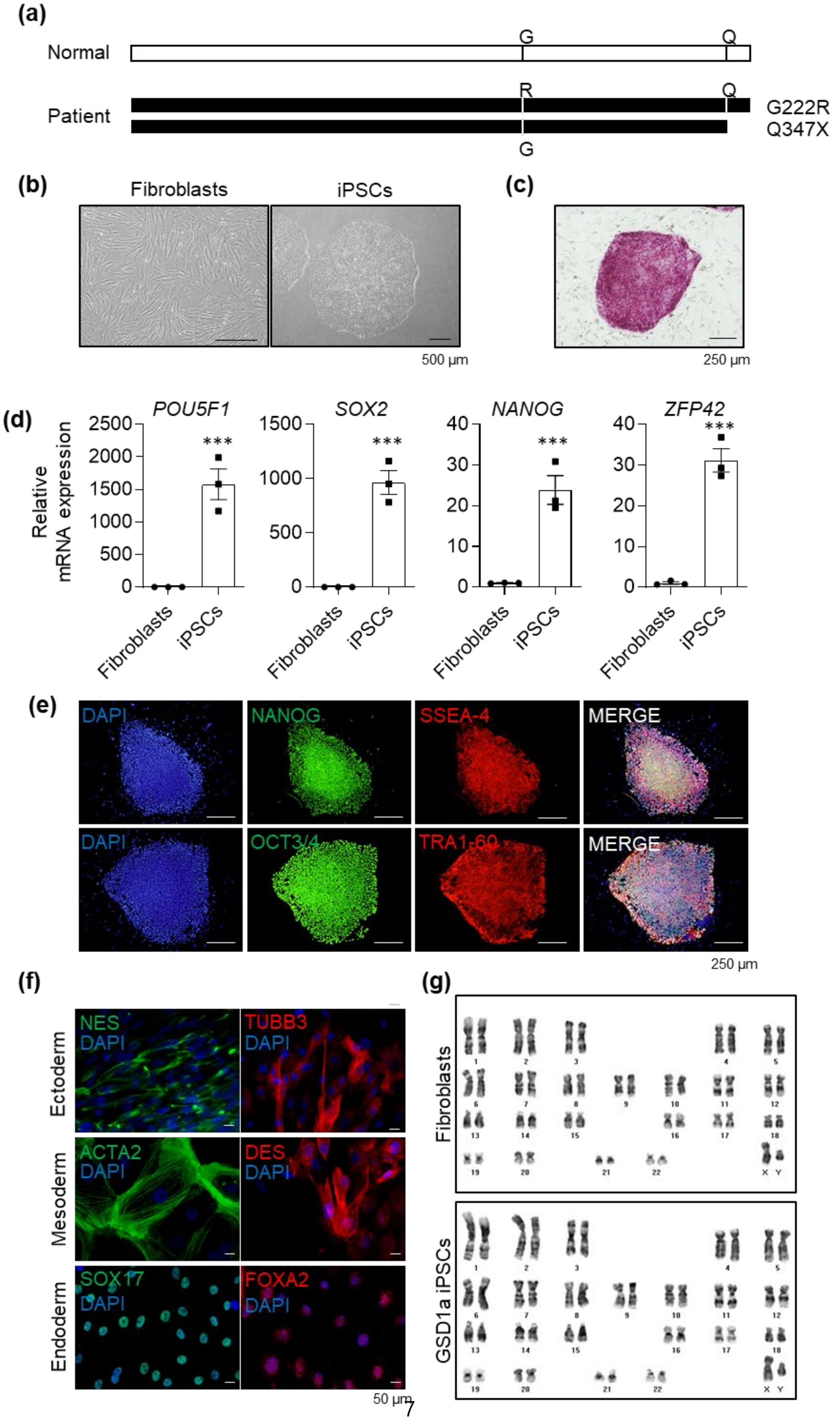

Supplemental Figure 8

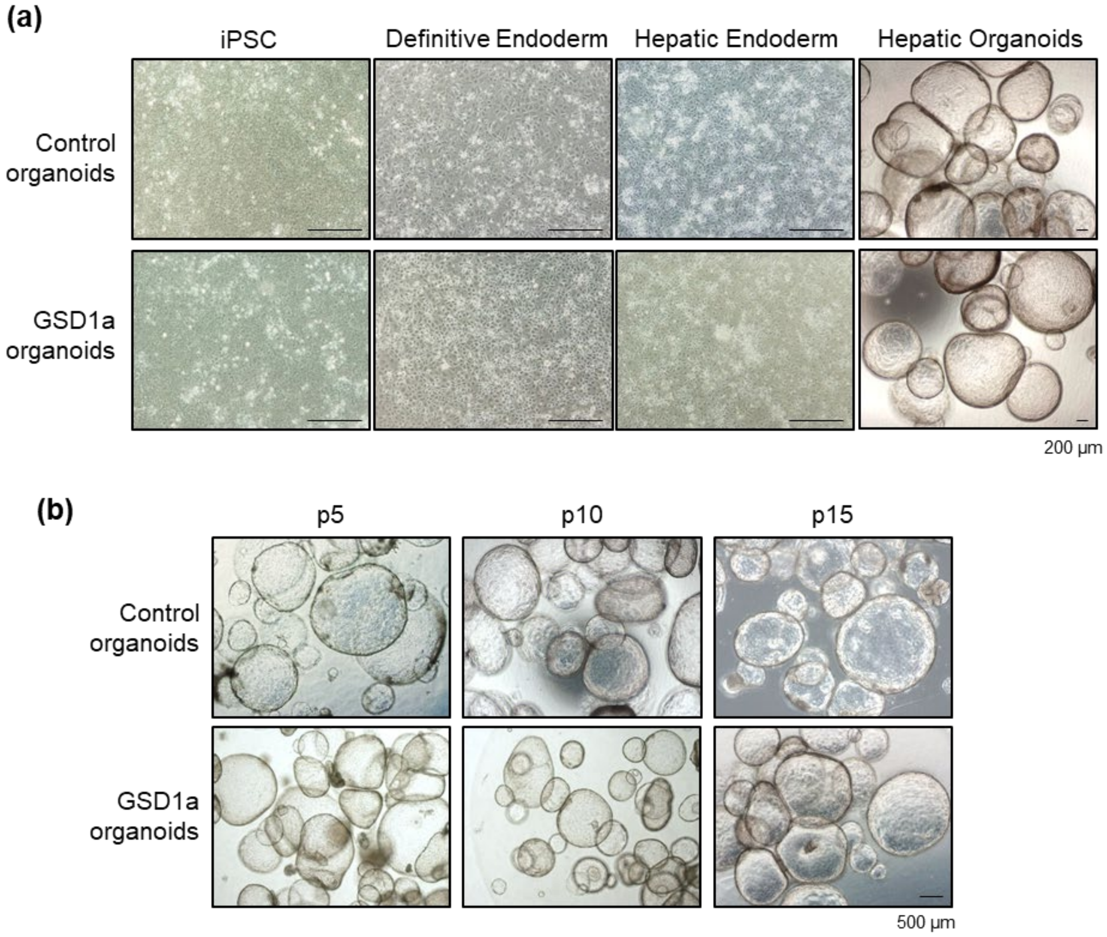

Supplementary Figure Legends

Figure S1. mRNA expression levels of the indicated genes in the HM (C) condition from passage 0 (p0) to passage 10 (p10). Data are the mean ± SEM (n = 3) and analyzed by the Student's t-test. *p < 0.05, **p <0.01, and ***p < 0.001.

Figure S2. Differentially expressed genes (DEGs) and pathways in the HM condition compared with the EM condition. **(a)** Volcano plot depicting DEGs in HM compared with EM. **(b)** The top-ranked hallmark enriched subset signatures of organoids in HM compared with EM in GSEA.

Figure S3. Essential factors in the medium for long-term culture. **(a)** Representative morphology of late passage organoids in conditions #1 and #8 at 6 weeks after factor depletion. **(b)** Percentage of small organoids in each condition at 6 weeks. Data are the mean ± SEM (n = 3) and analyzed by the Student's t-test. *p < 0.05 and ***p < 0.001.

Figure S4. Molecular characteristics of proliferative liver organoids. **(a)** The top-ranked hallmark enriched subset signatures of organoids in HM compared with MH in GSEA. ● : functional maturation-related pathways, ● : proliferation-related pathways. **(b)** Enrichment plot (*each left*), heat map (*each middle*), and list of representative gene sets (*each right*) enriched in HM compared with MH. **(c)** The top-ranked 2-fold upregulated genes in HM compared with MH among DEGs. **(d)** The top ten ranked biological processes are determined by GO analysis. **(e)** mRNA expression levels of genes related to lipid and bile acid metabolism in MH and HM condition. Data are the mean ± SEM (n = 3) and analyzed by the Student's t-test. *p < 0.05 and **p < 0.01.

Figure S5. Effects of FGFR4 knockdown during organoid generation. **(a)** Organoid size of shNT- and shFGFR4-transduced cells at 7 days after transduction. mRNA expression levels of genes related to **(b)** FGFR signaling and **(c)** the cell cycle and hepatic stem cells in each condition at 7 days after knockdown. Data are the mean ± SEM (n = 3) and analyzed by the Student's t-test. *p < 0.05 and ***p < 0.001.

Figure S6. Characterization of the HM condition by transcriptome analysis. **(a)** List of genes expressed in HM but not in MH using the LiGEP, which contains 93 genes expressed only in adult human liver tissue [15]. **(b)** mRNA expression levels of the indicated genes in MH and HM conditions. Data are the mean ± SEM (n = 3) and analyzed by the Student's t-test. *p < 0.05.

Figure S7. Generation of iPSCs from a GSD1a patient's fibroblasts and their characterization. **(a)** Mutation sites of the GSD1a patient. **(b)** Morphology of the patient's fibroblasts and iPSCs generated by reprogramming of these fibroblasts using Sendai virus. **(c)** Representative AP staining image of the patient's iPSCs. **(d)** mRNA expression levels of stemness markers in the patient's fibroblasts and iPSCs. **(e)** Immunostaining of stemness markers in the patient's iPSCs. **(f)** Immunostaining of markers of the three germ layers in attached EBs after spontaneous differentiation of the patient's iPSCs. **(g)** Karyotype analysis of the patient's fibroblasts and iPSCs. Data are the mean ± SEM (n = 3) and analyzed by the Student's t-test. ***p < 0.001.

Figure S8. Generation of liver organoids from GSD1a patient-derived iPSCs using the new protocol. **(a)** Representative morphology at each stage of organoid generation from control (*upper*) and GSD1a patient-derived (*lower*) iPSCs. **(b)** Representative morphology of late

passage control (*upper*) and GSD1a (*lower*) organoids.

Table S1. Medium composition for liver organoids

Reagent	Company	Catalog No.	Working Conc.			
			MH	HM	EM	DM
Hepatocyte Culture Medium (without EGF)	Lonza	CC-3198	0.5x	-	-	-
Endothelial Cell Growth Medium-2	Lonza	CC-3162	0.5x	-	-	-
FBS	Thermo Fisher	26140-079	2.5%	-	-	-
Advanced DMEM/F12	Thermo Fisher	12634028	-	1x	1x	1x
Penicillin Streptomycin	Thermo Fisher	15140122	-	1%	1%	1%
GlutaMAX	Thermo Fisher	35050079	-	1%	1%	1%
HEPES	Thermo Fisher	15630080	-	10 mM	10 mM	10 mM
N2 supplement	Thermo Fisher	17502048	-	1x	1x	1x
B27 supplement without vitamin A	Thermo Fisher	12587010	-	1x	1x	-
B27 supplement with vitamin A	Thermo Fisher	17504044	-	-	-	1x

N-Acetylcysteine	Sigma-Aldrich	A9165	-	1 mM	1 mM	1 mM
[Leu15]-Gastrin I human	Sigma-Aldrich	G9145	-	10 nM	10 nM	10 nM
Recombinant human EGF	Peprotech	AF-100-15	-	50 ng/ml	50 ng/ml	50 ng/ml
Recombinant human HGF	Peprotech	100-39	10 ng/ml	25 ng/ml	25 ng/ml	25 ng/ml
A83-01	Tocris	2939	-	5 μM	5 μM	0.5 μM
Nicotinamide	Sigma-Aldrich	N0636	-	10 mM	10 mM	-
Forskolin	Sigma-Aldrich	F3917	-	10 μM	10 μM	-
Recombinant human R-spondin	R&D	4645-RS-025	-	-	1 μg/ml	-
Recombinant human FGF10	Peprotech	100-26	-	-	100 ng/ml	-
*Recombinant human FGF-basic	Peprotech	100-18B	-	10 ng/ml	-	-
*Oncostatin M	R&D	295-OM	20 ng/ml	10 ng/ml	-	-
*ITS	Thermo Fisher	41400045	-	5 μg/ml	-	-
Dexamethasone	Sigma-Aldrich	D4902	100 nM	100 nM	-	3 μM

DAPT	Sigma-Aldrich	D5942	-	-	-	10 µM
Recombinant human BMP7	Peprotech	120-03	-	-	-	25 ng/ml
Recombinant human FGF19	Peprotech	100-32	-	-	-	100 ng/ml

* HM medium only components

Table S2. A list of DEGs in HM compared with EM

Up-regulated gene list			
	Gene_id	FC	P-value
1	DUOXA2	3.376869	0.044202
2	PPP1R1B	3.278671	0.024865
3	B3GNT7	2.961428	0.048469
4	SGK1	2.693897	0.024719
5	CEBPD	2.587388	0.013879
6	SULF1	2.526197	0.042764
7	IFITM1	2.524463	0.031604
8	TSC22D3	2.516602	0.045312
9	FGG	2.491963	0.038361
10	HIF3A	2.469181	0.045768
11	SNCG	2.280358	0.03798
12	BCL3	2.225421	0.029063
13	IRS2	2.154637	0.027918
14	OPN3	2.120647	0.02357
15	NTS	2.10237	0.036479
16	INHA	2.095011	0.049563
17	ZNF467	2.084592	0.045977
18	KLF9	2.045197	0.012691
19	LOC645166	2.029289	0.006025
20	LOC100506100	1.98939	0.024228
21	ICAM1	1.967679	0.026541
22	VGF	1.957093	0.027113
23	ZFP36	1.949256	0.016903
24	TMEM163	1.925832	0.005462
25	TMEM176A	1.921391	0.020618
26	IGF2	1.891956	0.049382
27	INSR	1.880273	0.041124
28	HRASLS2	1.869973	0.037081

Down-regulated gene list			
	Gene_id	FC	P-value
1	SCARNA16	-4.0001	0.000836
2	KRT20	-3.63367	0.015624
3	CFTR	-3.18981	0.031111
4	HLA-DMB	-2.8637	0.018741
5	EPHA6	-2.69426	0.008348
6	FAM198B	-2.64689	0.016741
7	PRSS35	-2.56793	0.024249
8	PODXL	-2.56492	0.004592
9	CASC8	-2.26426	0.035771
10	BMP4	-2.22779	0.025406
11	LINC00239	-2.13933	0.048905
12	CD55	-2.09363	0.026306
13	AXIN2	-2.09024	0.033284
14	GLDC	-2.04118	0.012566
15	LOC102724094	-2.03475	0.037668
16	CTSC	-1.97287	0.021497
17	DLGAP1-AS2	-1.96173	0.034246
18	SYTL5	-1.87435	0.006237
19	VWA2	-1.83256	0.021784
20	CPN1	-1.80328	0.00631
21	GLYATL1	-1.74613	0.015316
22	PCCB	-1.5653	0.042544
23	ABCC4	-1.45178	0.027586
24	EML1	-1.44905	0.021768
25	GPR75-ASB3	-1.44837	0.016042
26	LCP1	-1.41779	0.037224
27	GSTA2	-1.41175	0.043465
28	PARD3B	-1.39862	0.029974

29	LOC101928837	1.849178	0.035948
30	TAPBP	1.840072	0.000157
31	PLAUR	1.839975	0.041487
32	FAM46B	1.836151	0.023523
33	CDKN2D	1.82161	0.049164
34	GPR64	1.819486	0.04243
35	EHD2	1.815995	0.035559
36	HLA-B	1.792599	0.032968
37	EBI3	1.790713	0.00704
38	TENC1	1.765087	0.035309
39	TMEM176B	1.749609	0.00999
40	VASN	1.738124	0.023433
41	MAF	1.722568	0.016193
42	ELF3	1.722088	0.040593
43	TMEM178A	1.713536	0.002433
44	CEBPB	1.703802	0.047693
45	MEGF6	1.70106	0.027184
46	UBE2L6	1.69388	0.009083
47	NECAB1	1.690816	0.008714
48	BIRC3	1.688177	0.001739
49	UBALD2	1.67413	0.040887
50	LTBP3	1.669603	0.019347
51	SMOC2	1.662327	0.035086
52	C2	1.649996	0.037715
53	ERLIN1	1.642208	0.045333
54	AGTR1	1.639792	0.039803
55	FAM43A	1.636592	0.005715
56	PRSS8	1.601866	0.003435
57	LIX1L	1.601053	0.031374
58	MAN1A1	1.585254	0.027842
59	ABCG1	1.584166	0.042894

29	ABLIM1	-1.38968	0.041969
30	LOC388942	-1.38607	0.046187
31	FGFBP1	-1.37976	0.01226
32	SLC7A5	-1.3509	0.007118
33	MYO5C	-1.34081	0.044305
34	MYLK	-1.33616	0.012518
35	DDX21	-1.3304	0.015509
36	FAM155A	-1.32741	0.03745
37	LOC100294145	-1.29906	0.03998
38	SIDT1	-1.29761	0.044425
39	TNFRSF11B	-1.25461	0.004633
40	EIF3C	-1.2406	0.023723
41	PNPT1	-1.23342	0.04847
42	NIPAL1	-1.23045	0.039566
43	DPP10	-1.22237	0.015138
44	NAT2	-1.19251	0.006665
45	GULP1	-1.18625	0.038531
46	HSPA4L	-1.18051	0.023454
47	WDR12	-1.1713	0.024837
48	MTHFD1	-1.16307	0.028027
49	NEFH	-1.16295	0.018238
50	LINC01245	-1.15901	0.024987
51	DPP10-AS1	-1.13974	0.026523
52	RNASE6	-1.12815	0.032418
53	BAMBI	-1.12538	0.017431
54	SLC26A2	-1.11833	0.030386
55	TSPAN7	-1.10673	0.010913
56	GART	-1.09681	0.013756
57	ANKH	-1.09569	0.019354
58	MARC1	-1.05981	0.029581
59	BOP1	-1.05701	0.039953

60	ZFYVE28	1.583578	0.034883
61	H19	1.583548	0.004188
62	SLCO3A1	1.579074	0.012122
63	RAB27A	1.577811	0.009399
64	VAMP8	1.577578	0.046041
65	PRSS22	1.570863	0.006374
66	RBPMS	1.569565	0.000544
67	SAA2	1.564855	0.013905
68	SCN1B	1.555038	0.039589
69	RBP4	1.549331	0.024839
70	DLL1	1.535003	0.047154
71	CSF3R	1.529041	0.027376
72	BCL6	1.527845	0.018551
73	IRF2BPL	1.527395	0.005556
74	MIA	1.526072	0.025194
75	RGS10	1.525441	0.022852
76	NOL3	1.516917	0.011935
77	IDUA	1.507025	0.000124
78	ETS2	1.50624	0.011836
79	LRP11	1.50576	0.006367
80	IRF7	1.504146	0.026967
81	LOC158960	1.502731	0.000666
82	NFKBIA	1.501696	0.034138
83	P4HA2-AS1	1.498696	0.047618
84	TMEM25	1.496829	0.031883
85	NAGLU	1.493061	0.038668
86	GPR115	1.492719	0.033154
87	GRIN3B	1.487728	0.037471
88	HK2	1.484736	0.00032
89	MGAT4A	1.483724	0.037105
90	MARVELD1	1.481981	0.007377

60	IDH1	-1.04762	0.036278
61	COL26A1	-1.03847	0.04892
62	COA7	-1.03701	0.009941
63	TPD52L1	-1.0325	0.009651
64	IL17RD	-1.0309	0.040842
65	APITD1-CORT	-1.0301	0.006456
66	SRI	-1.0179	0.047578
67	PTCD1	-1.01553	0.015541
68	RPL13AP20	-1.01534	0.042655
69	TMEM106C	-1.01391	0.046521
70	HIST1H4C	-1.00775	0.009277

91	APOC1	1.480114	0.032029
92	FLJ20021	1.480041	0.049174
93	PQLC3	1.478608	0.043884
94	PLAGL1	1.476969	0.020558
95	IL11RA	1.476949	0.002195
96	MMP24-AS1	1.475972	0.018547
97	ST6GALNAC6	1.475126	0.047059
98	LOC101928303	1.472565	0.020381
99	SLC50A1	1.464675	0.047708
100	SDC4	1.462906	0.044529
101	PRKCDBP	1.4613	0.02689
102	LINC01214	1.455848	0.046893
103	FXYD5	1.44952	0.003584
104	ERICH2	1.447093	0.045168
105	DUSP10	1.446499	0.03188
106	AKIP1	1.446325	0.019479
107	TP53I13	1.446212	0.008215
108	RELL1	1.4442	0.030349
109	CARHSP1	1.443612	0.024337
110	HLA-E	1.440885	0.046916
111	OBSL1	1.437945	0.024913
112	NEURL1	1.437818	0.04737
113	TFF3	1.436358	0.041721
114	CHST12	1.434499	0.021129
115	SPACA6P	1.434429	0.019726
116	PSORS1C1	1.430503	0.001418
117	PPL	1.427155	0.020324
118	IMPDH1	1.427122	0.006156
119	ARSA	1.427112	0.04701
120	ZDHHC14	1.425791	0.048648
121	RASSF4	1.425271	0.036538

122	TCIRG1	1.424946	0.042945
123	BTG1	1.424355	0.007957
124	HAMP	1.42244	0.029473
125	UPK3B	1.420089	0.038399
126	NXNL2	1.419872	0.049052
127	STAC	1.419131	0.006689
128	DYNLRB2	1.417846	0.036251
129	RGS17	1.416496	0.011608
130	LOC100862671	1.41424	0.022707

Table S3. A list of primer sequences

Gene	Primer (Forward)	Primer (Reverse)
ACTB	GGACTTCGAGCAAGAGATGG	AGCACTGTGTTGGCGTACAG
AFP	AGCTTGGTGGTGGATGAAAC	CCCTCTTCAGCAAAGCAGAC
ALB	TTTATGCCCCGGAACTCCTTT	AGTCTCTGTTTGGCAGACGAA
ALDH1A2	AGTGTTTTCCAACGTCACTGAT	AGTCTGAGTTATTGGCTCTTTCG
APOA2	CATGTGTGGAGAGCCTGGTT	GCAGGCTGTGTTCCAAGTTC
APOA4	CTCAAGGGACGCCTTACGC	GTCCTGAGCATAGGGAGCCA
CCND1	GCATGTTCGTGGCCTCTAAG	CGTGTTTGCGGATGATCTGT
CD44	CCAGAAGGAACAGTGGTTTGGC	ACTGTCCTCTGGGCTTGGTGTT
CDKN1A	CGATGGAACTTCGACTTTGTCA	GCACAAGGGTACAAGACAGTG
CDKN2A	CCCAACGCACCGAATAGTTA	ACCAGCGTGTCCAGGAAG
CEBPA	AGGAGGATGAAGCCAAGCAGCT	AGTGCGCGATCTGGAACTGCAG
CYP2C8	CATTACTGACTTCCGTGCTACAT	CTCCTGCACAAATTCGTTTTCC
CYP2C9	CTACAGATAGGTATTAAGGACA	GCTTCATATCCATGCAGCACCAC
CYP3A4	CTTCATCCAATGGACTGCATAAAT	TCCCAAGTATAACACTCTACACAGACAA
CYP39A1	AAACTCACCCAATTATGGGCTC	TGAGAAAGGACGTATGCAAGTG
CYP8B1	GAGGACAGCCTCTTTCGCTT	TGTAGCCGAACAAGCTCAGG
FABP1	GCAGAGCCAGGAAAACTTTG	TCTCCCCTGTCATTGTCTCC
FGFR3	TCCATCTCCTGGCTGAAGAACG	TGTTCTCCACGACGCAGGTGTA
FGFR4	GCACTGGAGTCTCGTGATGG	CCACAGCGTTCTCTACCAGG
FGFRL1	CACGTCCTTCCAGTGCAAG	GGCAGCACCACAAACTTCTG
F12	CTCTGTCCACAACACCTCACTG	ATCAGGACCCTTGCACTGGCAT

G6PC1	GGGAAAGATAAAGCCGACCTAC	CAGCAAGGTAGATTCGTGACAG
HNF4A	GGCCAAGTACATCCCAGCTTT	CAGCACCAGCTCGTCAAGG
KRT19	AGCATGAAAGCTGCCTTGGA	CCTGATTCTGCCGCTCACTATC
LGR5	GACTTTAACTGGAGCACAGA	AGCTTTATTAGGGATGGCAA
MAPK3	CTACACGCAGTTGCAGTACAT	CAGCAGGATCTGGATCTCCC
NANOG	GCAGAAGGCCTCAGCACCTA	AGGTTCCCAGTCGGGTTCA
PROM1	TCAGTGAGAAAGTGGCATCG	TACACGTCCTCCGAATCCAT
POU5F1	GCTCGAGAAGGATGTGGTCC	CGTTGTGCATAGTCGCTGCT
SERPINA6	AGCCATCCTCGTCCTGGTCAAC	CCTTCACCACAGTTGTCTCGTC
SLC2A2	ATGTCAGTGGGACTTGTGCTGC	AACTCAGCCACCATGAACCAGG
SLC38A4	TGTATGCCACCCTGAGGTCCTT	GCAGGTACATGACAAGCATCCC
SOX2	CACTGCCCCTCTCACACATG	TCCCATTTCCCTCGTTTTTCT
TAT	TGGAGTTCACGGAGCGGTTAGT	GGACTGTGATGACCACTCGGAT
TP53	GGAACTCAAGGATGCCCAG	CAAGAAGTGGAGAATGTCAGTC
TTR	TGGGAGCCATTTGCCTCTG	AGCCGTGGTGGAATAGGAGTA
ZFP42	GGAATGTGGGAAAGCGTTCGT	CCGTGTGGATGCGCACGT

Table S4. A list of antibodies

Antibodies	Catalog no.	Company	Dilution
anti-ACTA2	A5228	Sigma-Aldrich	1:200
anti-ALB	A80-129a	Bethyl Laboratories	1:100
anti-CASP3	9662S	Cell Signaling	1:100
anti-DES	AB907	Merck	1:50
anti-CDH1	610181	BD Biosciences	1:100
anti-FOXA2	07-633	Merck	1:100
anti-HNF4A	3113	Cell Signaling Technology	1:200
anti-MKI67	Ab15580	Abcam	1:100
anti-NANOG	AF1997	R&D Systems	1:40
anti-NES	MAB5326	Merck	1:100
anti-POU5F1	sc-9081	Santacruz	1:100
anti-SOX17	MAB1924	R&D Systems	1:50
anti-SSEA4	MAB4304	Merck	1:100
anti-TRA-1-60	MAB4360	Merck	1:50
anti-TUBB3	PRB-435P	BioLegend	1:500
